# An artefact of PET attenuation correction caused by iron overload of the liver in clinical PET-MRI

**DOI:** 10.1186/s41824-017-0015-x

**Published:** 2017-11-08

**Authors:** Florian Büther, Benjamin Noto, Katharina Auf der Springe, Thomas Allkemper, Lars Stegger

**Affiliations:** 1 Department of Nuclear Medicine, University Hospital Münster, Albert-Schweitzer-Campus 1, 48149 Münster, Germany; 2 European Institute for Molecular Imaging, University of Münster, Münster, Germany; 3 Institute for Clinical Radiology, University Hospital Münster, Münster, Germany

**Keywords:** PET-MRI, Attenuation correction, Image artefacts, Attenuation correction

## Abstract

**Background:**

Attenuation correction is one of the most important steps in producing quantitative PET image data. In hybrid PET-MRI systems, this correction is far from trivial, as MRI data are not correlated to PET attenuation properties of the scanned object. Commercially available systems often employ correction schemes based on segmenting the body into different tissue classes (air, lung tissue, fat-, and water-like soft tissue), e.g. by using a dual time-point Dixon sequence. However, several pitfalls are known for this approach. Here a specific artefact of MR-based PET attenuation correction is reported, caused by misidentifying the liver as lung tissue due to iron overload.

**Case presentation:**

A patient with a history of hematopoietic stem cell transplantation underwent a whole-body [^18^F]FDG PET-MRI scan. Markedly low liver uptake values were noted in the PET images, seemingly caused by an erroneous assignment of lung tissue attenuation values to the liver. A closer investigation demonstrated markedly low MRI intensity values of the liver, indicative of secondary hemochromatosis (iron overload) most probably due to a history of multiple blood transfusions. Manual assignment of adequate liver attenuation values resulted in more realistic PET images.

**Conclusions:**

Iron overload of the liver was identified as a cause of a specific attenuation correction artefact. It remains to be seen how frequent this artefact will be encountered; however, this case highlights that attenuation maps should always be checked during PET image interpretation in hybrid PET-MRI.

## Background

In order to obtain images of high visual quality and reliable quantification, measured PET emission data have to be corrected for several effects prior to or during reconstruction. Arguably the most important of these corrections is the correction for losses of released 511 keV photons due to attenuation effects (photo effect, Compton scattering) when traversing different tissues (attenuation correction, AC). If spatially-resolved information about the attenuation properties of the scanned object/subject is known, AC is a straight-forward problem that can be easily implemented.

In combined PET-CT systems, CT images are used for this task after scaling from x-ray energies of around 50–140 keV to 511 keV. However, the presence of high-*Z* elements (e.g., Barium or Iodine as components of CT contrast media) may result in a failure of correct scaling, potentially leading to quantitative errors and even pronounced artefacts in the corrected PET images (Büther & Schober, 2014).

Hybrid PET-MRI systems are now available for clinical imaging (Quick, 2014). As MR images are not related to attenuation properties for gamma radiation, more complex algorithms have to be applied in order to get reasonable estimates of PET attenuation maps (AM). Typically, these involve the usage of two-point 3D Dixon sequences to acquire in-phase and opposed-phase images, allowing the segmentation of the body in fat- and water-like tissues (Martinez-Möller et al., 2009). Additionally, air and lung tissue can be identified as regions possessing very low MR signals. Lung tissue can then be segmented by a connected component analysis (Hofmann et al., 2011). Reasonable PET attenuation values for water-, fat-, and lung-like tissues as well as air are then assigned to each determined compartment.

There are however several known limitations with this approach (Wagenknecht et al., 2013; Keller et al., 2013). For example, bone attenuation is difficult to implement into Dixon-based AM as bones are not readily identifiable on standard MR images. Furthermore, the transaxial MRI field-of-view is usually limited and does not cover the whole gantry opening, leading to truncation of attenuation data which is a problem especially when patients are scanned with their arms next to their body (Rausch et al., 2016). In some cases the segmentation into water- and fat-like tissues fails, leading to fat-water inversion of attenuation values, again potentially resulting in incorrect PET image values (Rausch et al., 2016).

A recent study demonstrated that iron-containing MR contrast agents may pose a problem in Dixon-based attenuation correction for PET/MRI since iron heavily influences relaxation times of proton spins (Borra et al., 2015). The authors could show that liver attenuation was severely underestimated in a baboon study, resulting in artificially decreased liver SUV. We here report the occurrence of a similar type of attenuation correction artefact related to the presence of hemochromatosis in a clinical [^18^F]FDG PET-MRI study.

## Case presentation

A 49-year-old patient with a history of acute myeloid leukaemia and subsequent hematopoietic stem cell transplantation (five weeks prior to the scan) underwent a whole-body [^18^F]FDG PET-MRI scan in order to investigate a suspected gastrointestinal graft-versus-host disease (GVHD). This was performed on a Biograph mMR system (Siemens Healthineers, Erlangen, Germany) one hour after i.v. injection of 275 MBq [^18^F]FDG. Scanning was performed in a supine position with the arms lying next to the patient’s torso. Dedicated RF coils, either being corrected for in PET AC by templates (mMR head/neck coil, mMR spine coil) or optimised for minimal PET attenuation (mMR body coils), were used for MR imaging (total imaging matrix technology, Siemens Healthineers, Erlangen, Germany). A standard two-point Dixon sequence (pre-Gadolinium) was performed for AM generation, resulting in AM images of four different tissue classes (air, lung, fat-, and water-like soft tissue). Due to transaxial MR field-of-view truncation, attenuation of the arms was estimated using PET emission data by applying the maximum likelihood reconstruction of activity and attenuation algorithm (MLAA). PET imaging was performed using 6 bed positions with acquisition durations of 3 min for each bed position. PET raw data were corrected for randoms, scatter, and attenuation, and reconstructed using a 3D ordinary Poisson ordered subsets expectation maximisation (OP-OSEM) algorithm with 3 iterations and 21 subsets, followed by a 5 mm full-width-at-half-maximum (FWHM) Gaussian filter. Acquired MR data involved transaxial T1-weighted VIBE and T2-weighted HASTE sequences as well as coronal T1-weighted VIBE sequences post-Gadolinium.

**Fig. 1:**
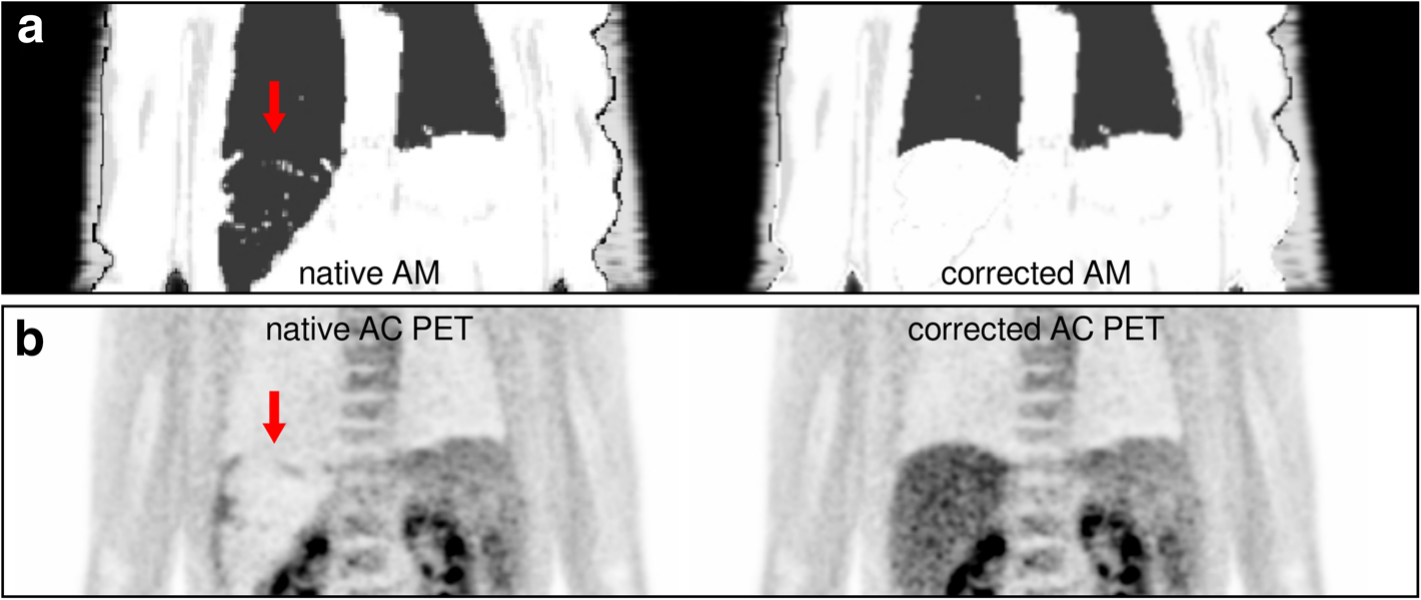
Coronal slice of the MRI-derived attenuation maps (AM; **a**) and attenuation-corrected [^18^F]FDG PET images (AC PET; **b**) demonstrating misidentification of liver tissue as lungs and artificially suppressed tracer uptake in the liver (arrows). Native data are shown on the left side while manually-corrected attenuation map and resulting PET data are shown on the right side

**Fig. 2:**
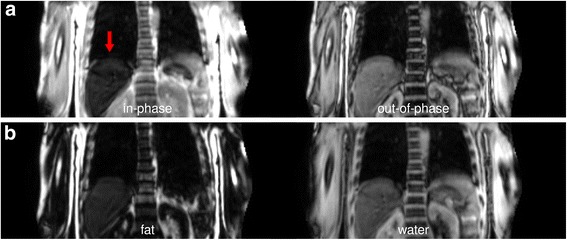
Coronal slice of in-phase and out-of-phase Dixon MR images (**a**) and determined fat and water images (**b**). The in-phase image demonstrates markedly low MR signals of the liver (arrow)

Inspection of the determined AM demonstrated that most parts of the liver (approximately 82% as determined by volumetric analysis of MR and AM images) had not been identified as water-like soft tissue (with assigned linear absorption coefficients of 0.1000 cm^−1^), but as lung tissue (0.0224 cm^−1^) (Fig. 1a). This in turn led to highly suppressed PET activity values of the liver in the reconstructed images with an apparent SUV_mean_ of the liver amounting to 0.6, far outside a typical reference range of 1.4–3.2 (Boktor et al., 2013) (Fig. 1b). The acquired Dixon sequence-based in-phase image (Fig. 2a) demonstrated markedly reduced signal intensities of the liver, similar to those of lung tissue. This translated into comparatively low values in the calculated water image (Fig. 2b). As the liver is located right next to lung tissue, it was misinterpreted as lung since the gradient of image values between lung and liver was not large enough for the connected component segmentation algorithm to handle this properly.

The loss in liver signal intensity was also clearly visible in the T1- and T2-weighted images, and was retrospectively attributed to secondary hemochromatosis caused by a history of multiple blood transfusions due to acute myeloid leukaemia. This led to elevated levels of iron in the liver (iron overload). Due to their superparamagnetic properties, these iron deposits caused increased relaxations of proton spins, hence suppressing MR signals (Queiroz-Andrade et al., 2009).

**Fig. 3:**
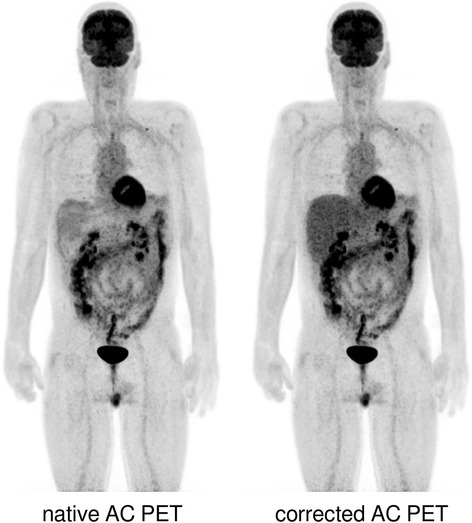
Maximum intensity projections of native (left) and corrected whole-body PET (right), demonstrating elevated tracer uptake levels in the rectum, the sigmoid colon, as well as the ascending and descending colon

PET images revealed elevated tracer uptake levels in the rectum, the sigmoid colon, as well as the ascending and descending colon (Fig. 3), indicative of GVHD in accordance with a study by Stelljes et al. (Stelljes et al., 2008). However, as tracer uptake in the liver serves as a major reference in [^18^F]FDG PET-based diagnosis of gastrointestinal GVHD (Stelljes et al., 2008), a more detailed qualitative assessment of GVHD beyond simple SUV determination proved to be difficult in this case.

Manual segmentation of the lung-liver border in the AM along the visible border in the Dixon water image (Fig. 2b), assigning a soft-tissue value of 0.1000 cm^−1^ (Fig. 1a), and reconstructing the PET data with this modified AM resulted in PET images with visually typical [^18^F]FDG uptake in the liver (Fig. 1b, Fig. 3). Liver SUV_mean_ then amounted to 3.1, well within a normal range of values.

## Discussion and conclusion

Secondary hemochromatosis (iron overload) of the liver following multiple blood transfusions was found to be the cause of an attenuation correction artefact in PET-MRI. The loss of MR signal intensity in the in-phase Dixon images, caused by superparamagnetic iron deposits, led to problems in identifying the border between lung tissue and the liver and a subsequent erroneous assignment of lung attenuation values to the liver. Attenuation-corrected PET demonstrated highly suppressed liver uptake levels, making a reliable semi-quantitative PET-based evaluation of GVHD impossible. A manual segmentation of the liver in the AM, assigning a realistic linear absorption coefficient to the liver, led to more conventional tracer uptake values in the liver.

To our knowledge, this is the first time that hepatic iron overload has been described as a cause of PET attenuation artefacts in combined clinical PET-MRI. Considering the numerous potential pitfalls related to MRI-based attenuation correction and the relative rarity of this artefact, it might not seem to be of paramount urgency to develop specific corrections (this being also the reason why a manual correction of attenuation values was chosen here). However, small adjustments to the lung segmentation algorithm may be sufficient to alleviate this type of artefact, as there is still a small gradient between lung and liver visible in the Dixon-based fat and water images (Fig. 2).

Alternatively, attenuation maps derived from ultrashort echo time sequences (uTE) may help to better delineate the border between lung and liver as demonstrated in a baboon study in order to measure MR signals at times when proton spins have not yet relaxed (Borra et al., 2015). Unfortunately, no uTE scans for AM generation were performed in our study, as this is a standard procedure only for head scans at our department and potentially leads to unwanted additional artefacts. In this respect, a combination of both Dixon- and uTE-based AM derivation may prove to be a very robust method to determine accurate attenuation information (Berker et al., 2012).

Finally, this case once again highlights the necessity of always inspecting the determined AM together with PET images with and without attenuation correction in order to identify attenuation correction artefacts that may not be obvious at first sight.

## Abbreviations

AC: Attenuation correction
AM: Attenuation map
CT: Computer tomography
FDG: Fluorodeoxyglucose
GVHD: Graft-versus-host disease
HASTE: Half fourier acquisition single shot turbo spin echo
MLAA: Maximum likelihood reconstruction of activity and attenuation
MR: Magnetic resonance
MRI: Magnetic resonance imaging
PET: Positron emission tomography
SUV: Standardised uptake value
uTE: Ultrashort echo time
VIBE: Volumetric interpolated breath-hold examination
